# The Catgut Ligature

**Published:** 1881-05

**Authors:** 


### Selections.

---

### THE CATGUT LIGATURE.

Prof. Lister delivered an Inaugural Address before the Clinical Society of London on the Preparation and Uses of the Catgut Ligature in Surgical Practice, from which we make copious extracts:

The catgut ligature has in some respects exceeded my original hopes. I feared that its advantages would be limited to wounds in which putrefaction was avoided, and that if septic suppuration took place in a wound in which it was employed for securing the vessels, the ligatures would sooner or later come away like little sloughs. Such, however, has not proved to be the case. Whatever be the progress of the wound, we never see anything of the catgut, so that even surgeons who have not adopted strict antiseptic treatment have been led to employ the new material in ordinary wounds. Under other circumstances, however, the catgut has often led to disappointment. We hear of cases in which the Cæsarean section has been performed, and all has gone on well until the knots of the catgut with which the uterine wound was secured have given way, and the patient's death has been the result. Again, in ligature of large arterial trunks in their continuity several surgeons have met with bitter disappointment, the case ending in disaster from secondary hemorrhage, or the treatment proving abortive through the channel of the vessels becoming opened up again, at the site of ligature. Hence many surgeons have been induced to return to silk, even though using strict antiseptic treatment; rendering the silk aseptic by steeping it in a suitable lotion, and cutting the ends short. This practice has, however, by no means proved uniformly successful. As an instance of unsatisfactory result I may mention a case recorded by Mr. Clutton in the last volume of our Transactions. He tied the external iliac artery, under strict antiseptic precautions, and the wound healed within a week; but, as I learned from a letter which he was good enough to send me at the time, "six weeks after the operation a little blister formed, and fluid began to escape, forming a small scab, and in three months the loop which had been placed around the artery came away." Such a result was not at all surprising to me, seeing that what induced me to try the animal ligature was the discovery of a small abscess about the remains of a partially absorbed silk thread, which I had applied in the same manner as Mr. Clutton, and, as it happened, to the same artery. It can hardly be doubted that suppuration proceeding from the immediate seat of the ligature, must be a source of danger. As an illustration of the mischief which a ligature of ordinary material may do, I may mention a case of goitre in a young woman on whom I operated on Jan. 28th of last year. It was of moderate dimensions, but the effect on the respiration was so considerable, that I determined to remove it, following Dr. Patrick Heron Watson's plan of preliminary deligation of the thyroid vessels circumferentially to the tumor. If this is effectually done, the operation is bloodless—so that as the laryngoscope applied by Dr. Felix Semon, who had recommended the case to my care, showed that the anterior wall of the trachea was pressed back considerably by the growth, I adopted a measure which I believe would in all cases of the removal of the thyroid prove advantageous—namely, I divided the tumor in the first instance in the middle line, so as in the event of adhesion to the trachea to be able to remove the two halves of the growth at leisure, dissecting it off from the trachea more or less completely as might be desired, leaving some portions at the adherent parts, so as to avoid the deadly risk of perforation of the air-passage. But in order that the circumferential ligature of the thyroid vessels may be secure, it is essential that the material should be very strong, so that the tissues round about the tumor, including the vessels, may be thoroughly tightened up. I possessed no catgut which I felt was strong enough to bear the full strength of my hands, and therefore I was compelled to use a hempen ligature, after, of course, carefully rendering it aseptic by means of the carbolic lotion. Six of these hempen ligatures were used, three on each side.

During the first eight days everything went on in a typical fashion according to the antiseptic method. There was a merely serous effusion rapidly diminishing, and we looked to the wound being healed in a few days more. But on the ninth day there was seen to be a little something of purulence mingled with the discharge; and the pus afterwards became thicker, though always in small quantity; a little could be pressed out from each side, and in a month one of the hempen ligatures made its escape. Five days later four others of the hempen threads came away, altogether unaltered, as they may be seen on one of the cards on the table where I have exhibited them. I submitted them to careful examination. They had a sour odor, and, applied to litmus paper, gave an acid reaction—that is to say, the natural alkaline condition of the blood serum had been changed to acidity by some peculiar species of fermentation. On examining them with the microscope I found the interstices of the threads of the hemp loaded with a little organism, to which I believe I happened to be the first to direct attention as to its mode of growth, and to which I gave the name of Granuligera, occurring in groups of two, three, four, and so forth, as distinguished from the chains of ordinary bacteria, and of which one form at least has been since shown by Mr. Cheyne to occur very frequently in cases treated antiseptically without any interference with aseptic progress. I found that the interstices of the threads of the hemp were loaded with these little micrococci. It so happens I have had the opportunity within the last few days of obtaining a sample of these micrococci, thanks to Mr. Cheyne's kindness. He brought this flask of a pure and perfectly transparent infusion of meat to a case which I had operated on a fortnight before by excision of the ankle. The skin had been unbroken, so that I was able to operate antiseptically, and the case pursued a perfectly typical course. The wounds, which were left gaping at the time of the operation, were filled with blood-clot, which remained unaltered in appearance, though undoubtedly organized by that time, more or less. A little piece of the blood-clot from one of these wounds was introduced with careful antiseptic precautions into the flask of clear fluid, and you see it is now turbid; and there is under the microscope on the table a specimen of the little organism to which the turbidity is due. But though, under ordinary circumstances, these micrococci may be present, as Mr. Cheyne has abundantly shown, and as the excision of the ankle I have just referred to illustrates, without causing any evil, yet there may be circumstances in which they may prove mischievous, and the case of goitre which I have been relating appears to have been one of these. The micrococci developing for a protracted period in the interstices of the hempen ligature produced an acid fermentation of the serum in its most aggravated form. The acid serum became a cause of irritation, and thus the ligatures, which otherwise, being unirritating in their own substance, might have become encapsuled, and in due time absorbed, became causes of suppuration. One of the six ligatures still remained unaccounted for. In due time we sent the patient home with a small sinus remaining, a little pus always discharging from it, but it was not until the middle of September that the last ligature came away altogether unaltered. Now, there is no doubt whatever that if I had had catgut which I could have trusted for the operation, the catgut ligatures would have been disposed of within two or three weeks, and the healing, instead of requiring eight months, would probably have been completed in a fortnight. Here, then, we have an illustration of the great disadvantage which may arise even under antiseptic treatment from the use of the ordinary forms of ligature.

The method of preparing catgut, which I published long ago, answers the purpose very well even for the ligature of arteries in their continuity, provided certain conditions in its preparation be complied with. Such at least is my own experience. This, indeed, has not been very extensive, but it has been sufficient to deserve consideration. I have tied altogether nine large arteries in their continuity with prepared catgut. Of these one was a case of ligature of the carotid in a young woman aged twenty-two, with a pulsating tumor below the angle of the jaw, in the situation of a carotid aneurism and with all the symptoms of that disease. The application of the ligature reduced somewhat the pulsation and the dimensions of the swelling, but the further cure which we hoped for did not take place. She left the hospital with a pulsating tumor, and I heard only yesterday from the medical man under whose care she is in Scotland, that this tumor, for which I tied that carotid artery in 1874, still exists as a pulsating swelling, if anything rather on the increase. But though as regards the cure of the disease the ligature was unsatisfactory, nothing could be more beautiful in its effect as respects the healing of the wound without suppuration, and permanent obstruction of the artery at the seat of ligature.

As regards the mode of applying the ligature, I have always used a single reef knot, with short-cut ends, tying it sufficiently tightly to cause the giving way of the internal and middle coats. This latter point is not, indeed, essential; as I long ago surmised, and as Mr. Barwell's experience has demonstrated. But if, as in the case of catgut, the form of the ligature admits of it, the injury done to the deeper tunics is, I believe, advantageous, by leading to a salutary corroborative process of repair.

Why, it may naturally be asked, has my own experience been more satisfactory with the catgut ligature than that of many other surgeons? There are, I believe, two reasons for this: one is, that I have never ventured to tie an artery of considerable size in its continuity without having taken pains to ascertain that the catgut was of thoroughly trustworthy material; and the other reason is, that I have adopted strict antiseptic means of treatment, not only during the earlier stages of the case, but to the last. So long as any part of the wound remains unhealed, antiseptic treatment of the strictest kind ought, I believe, to be employed. Even though the sore may seem to be superficial, there may still exist a sinus leading down to the site of the ligature, and if ordinary treatment, as distinguished from antiseptic, be employed, down this sinus the septic process may advance and invade the ligature, and, inducing unhealthy suppuration and ulceration, may lead at last to disaster from hemorrhage. I know that this has actually taken place.

The method of preparation which I have now the honor to bring before you is the following: I dissolve one part of chromic acid in 4000 parts of distilled water, and add to the solution 200 parts of pure carbolic acid, or absolute phenol. In other words, I use a one-to-twenty watery solution of carbolic acid, only that the carbolic acid is dissolved, not in pure water, but in an exceedingly dilute solution of chromic acid. But minute as is the quantity of the chromic acid, it exerts, when in conjunction with carbolic acid, a most powerful effect upon the gut. The first effect of the addition of the carbolic acid to the chromic solution is to change its pale yellow color to a rich golden tint. But if the liquid is allowed to stand without the introduction of the catgut, it changes in the course of a few hours to a dingy reddish brown, in consequence of some mutual reaction of the two acids, and a considerable amount of gray precipitate is formed. If, however, catgut about equal in weight to the carbolic acid is added as soon as the ingredients, are mixed, the liquid retains its brightness, and the only change observed is a gradual diminution of the depth of the yellow color; the precipitate, which I presume still occurs, taking place into the substance of the catgut. As soon, therefore, as the preparing liquid, has been made, catgut equal in weight to the phenol is introduced into it. If you have too large a proportion of catgut, it will not be sufficiently prepared; if you have too small a quantity, it may run the risk of being over-prepared. At the end of forty-eight hours, catgut steeped in such a solution is sufficiently prepared. It is then taken out of the solution, and dried, and when dry is placed in one-to-five carbolic oil; it is then fit for use. I have here a sample of catgut prepared by this method. Although it has been steeped in warm blood serum since this morning at 11 o'clock, it is still translucent and firm, without being rigid, and a reef knot tied upon it holds with the most perfect security.—*London Lancet.*

